# Variation in Soil Microbial Community Structure Associated with Different Legume Species Is Greater than that Associated with Different Grass Species

**DOI:** 10.3389/fmicb.2017.01007

**Published:** 2017-05-31

**Authors:** Yang Zhou, Honghui Zhu, Shenglei Fu, Qing Yao

**Affiliations:** ^1^College of Horticulture, South China Agricultural University, Guangdong Province Key Laboratory of Microbial Signals and Disease Control, Guangdong Engineering Research Center for Litchi, Guangdong Engineering Research Center for Grass ScienceGuangzhou, China; ^2^State Key Laboratory of Applied Microbiology Southern China, Guangdong Provincial Key Laboratory of Microbial Culture Collection and Application, Guangdong Institute of MicrobiologyGuangzhou, China; ^3^College of Environment and Planning, Henan UniversityKaifeng, China

**Keywords:** legume species, grass species, soil microbial community structure, variation within taxonomical group, root exudates

## Abstract

Plants are the essential factors shaping soil microbial community (SMC) structure. When most studies focus on the difference in the SMC structure associated different plant species, the variation in the SMC structure associated with phylogenetically close species is less investigated. Legume (Fabaceae) and grass (Poaceae) are functionally important plant groups; however, their influences on the SMC structure are seldom compared, and the variation in the SMC structure among legume or grass species is largely unknown. In this study, we grew three legume species vs. three grass species in mesocosms, and monitored the soil chemical property, quantified the abundance of bacteria and fungi. The SMC structure was also characterized using PCR-DGGE and Miseq sequencing. Results showed that legume and grass differentially affected soil pH, dissolved organic C, total N content, and available P content, and that legume enriched fungi more greatly than grass. Both DGGE profiling and Miseq-sequencing indicated that the bacterial diversity associated with legume was higher than that associated with grass. When legume increased the abundance of *Verrucomicrobia*, grass decreased it, and furthermore, linear discriminant analysis identified some group-specific microbial taxa as potential biomarkers of legume or grass. These data suggest that legume and grass differentially select for the SMC. More importantly, clustering analysis based on both DGGE profiling and Miseq-sequencing demonstrated that the variation in the SMC structure associated with three legume species was greater than that associated with three grass species.

## Introduction

Soil ecological processes are primarily driven by soil microbial community (SMC), and plant species is one of the important factors shaping the SMC (Berg and Smalla, 2009; Diouf et al., 2010; Ladygina and Hedlund, 2010; McLaren and Turkington, 2011). When most studies focus on the difference in the SMC associated with different species, few study addresses to what extent the difference is among different species. Plants within a closely related taxonomic group are likely to share a number of common traits, e.g., root biomass, amount, and availability of the rhizodeposits, root defensive strategies, which are the key factors shaping the SMC. Therefore, Berg and Smalla (2009) pointed out that plant phylogenetic positions can have an influence on the SMC. Recently, Bouffaud et al. (2014) demonstrated that the difference in the SMC correlated significantly with the phylogenetic distance of their plant hosts, opening a novel perspective regarding the interactions between plants and the associated SMC.

Legume (Fabaceae) and grass (Poaceae) are the two most functionally important plant groups on the globe (Croser et al., 2006; Liu et al., 2009). In agroecosystems, legume, and grass normally occur as food crops, vegetables, forages, cover crops, and weeds. Due to their wide distribution in the diverse ecosystems, the influences of legume and grass on soil ecological processes have been intensively investigated, particularly the influences on the biogeochemical cycling of nutrients. Dinesh et al. (2006) reported that long-term (12 years) cover cropping with four leguminous species significantly improved the N and C cycling driven by soil microbes, resulting in higher levels of total organic C, dissolved organic C and N, labile organic N, and etc. Cui et al. (2015) indicated that, as cover crops in a subtropical orchard, legume and grass differentially affected the hydrolysis of soil organic P.

Since the SMC is central to the nutrient biogeochemical cycling and soil fertility, better understanding of the difference in the SMC between legume and grass is particularly necessary. Many environmental factors are involved in shaping the SMC, among which plant species is one of the most intensively studied factors (Diouf et al., 2010; Ladygina and Hedlund, 2010; McLaren and Turkington, 2011). In this context, it is reasonable to speculate that legume and grass can shape respectively specific SMC, which is supported by previous reports. For example, the SMC of legume (*Lotus corniculatus*) was much different from that of grass (*Holcus lanatus*) as revealed by PLFA (Ladygina and Hedlund, 2010). In more detail, with library construction and cloning, Chen et al. (2014) showed that legume (*Trifolium repens*) greatly enriched alpha-proteobacteria abundance, but reduced beta-proteobacteria abundance, indicating the improved soil fertility. For fungal taxa, Benitez et al. (2016) demonstrated that two legume cover crops (*Trifolium incarnatum* and *Vicia villosa*) preferably enriched arbuscular mycorrhizal fungi. Similar results were also reported by Turner et al. (2013), who employed metatranscriptomics technique. Collectively, these studies suggest that legume and grass tend to select specific microbial taxa, which is probably beneficial to themselves (Bakker et al., 2013). Notwithstanding, few studies have been performed to date to strictly compare the differential effects of legume and grass on the SMC, especially with more plant species, and general understanding of the effects of plant taxonomic groups on their associated SMC is still poor.

Cover cropping in orchards is world widely practiced for the sustainable production, where either legume or grass cover crops is commonly grown (Cui et al., 2015). This special agroecosystem provides ideal platform for the comparison of legume and grass regarding their effects on the SMC. We hypothesized that legume and grass differentially affect the SMC, and that the species within legume or grass may differ in their effects on the SMC. In this study, we constructed mesocosms using the orchard soils to grow three legume species vs. three grass species. We monitored the soil chemical properties, quantified the abundance of bacteria and fungi, and characterized the SMC using DGGE and Miseq-sequencing. By comparing the difference between legume and grass and the variation within three species of legume or grass, we aimed to explore (a) what differences in the SMC shaped by legume and grass, and (b) to what extent the SMC varies within legume or grass species.

## Materials and methods

### Plant species and soils

To compare the influences of legume and grass on the SMC, three legume species [*Stylosanthes guianensis* (Aubl.) Sw., *Trifolium pratense* L., *Medicago sativa* L.] and three grass species (*Paspalum natatum* Flüggé, *Festuca arundinacea* L., *Lolium perenne* L.) were used. We selected these plant species, because each of them belongs to a different genera and all of them are commonly used as cover crops in orchards. The seeds were commercially obtained (Yifang Seed Lt. Company, Jiangsu, China).

The soils were collected from a subtropical orchard (E 112°54′19″, N 22°40′20″). The orchard was transitioned from forest land and managed for over 30 years (Wang et al., 2015), and the understory of the forest was dominated by *Dicranopteris dichotoma* with the coverage as high as almost 100% (Zhao et al., 2012). The soil at 0~20 cm were collected and sieved through a 5 mm pore-sized mesh for late use. The soil chemical properties were analyzed as follows: pH 5.42, organic matter content (SOM) 1.04%, total N content (TN) 0.87 g kg^−1^, total P content (TP) 0.89 g kg^−1^, total K content (TK) 15.90 g kg^−1^, available N content (AN, alkali-hydrolyzable N) 67.4 mg kg^−1^, available P content (AP) 161.0 mg kg^−1^, available K content (AK) 72.6 mg kg^−1^.

### Experimental design and sampling

Plastic pot (32 cm length × 18 cm width × 13 cm height) was used as container to construct a mesocosm containing 4.5 kg soils. Seven treatments responding to six plant species plus a control without plants were set up, with each treatment comprising four replicates. In each pot with plants, surface disinfected (10% H_2_O_2_ for 15 min) and pre-germinated seeds were sown, and then 50 seedlings were left over at 10 days after sowing. We chose this planting density much higher than that in orchards, in order to produce high root density in soils and thus to reinforce the influence of plants on the SMC. The pots were placed in glasshouse with natural day/night photoperiod and temperature (22~35°C), and watered to the moisture of 20% every 3 days.

Soils were sampled at 160 days after sowing. At harvest, the shoots were firstly removed, and then the top soil layer of about 1 cm were discarded to avoid the aboveground disturbance. All roots (including visible root fragments) in the soils were taken off with forceps. The leftover soils were homogenized with a 2 mm pore-sized mesh and divided into two aliquots. One aliquot was air dried for chemical analysis, the other aliquot was stored at −80°C for molecular analysis.

### Soil chemical analysis

Soil pH was determined with deionized water (2.5:1, w/v) using a glass electrode (Sartorius PB-10). Total organic C (TOC) and dissolved organic C (DOC) were analyzed by titration after wet oxidation with H_2_SO_4_ and K_2_Cr_2_O_7_ (Heanes, 1984). TN, TP, and TK were measured using the Kjeldahl method, the molybdenum blue colorimetric method and the flame photometric method, respectively (Kirk, 1950; Sommers and Nelson, 1972). AN, AP, and AK were quantified by alkali-hydrolyzed reduction diffusing method, colorimetric method and flame photometric method, as described previously (Xiong et al., 2008).

### DNA extraction and PCR-DGGE

Soil total DNA was extracted using PowerSoil® DNA Isolation kit (MoBio Laboratories Inc.) following the manufacturer's protocol (Cui et al., 2014). To obtain sufficient amount of DNA and diminish bias of DNA extraction, 3 sub-samples were used for DNA extraction respectively, then put the obtained DNA together to get a composite DNA sample. PCR-DGGE of bacterial 16S rRNA gene and fungal ITS gene were performed with these DNA solutions.

PCR amplifications were conducted in triplicate for each of the soil samples. Nested PCR was conducted with 27F/1492R and GC-341F/518R as the first and second primer set to amplify the V3 region of bacterial 16S rRNA gene (Cui et al., 2014). Similarly, to amplify the fungal ITS1 gene, ITS1F/ITS4 and GC-ITS1F/ITS2 were used as the first and second primer set according to Anderson et al. (2003). Then the PCR products were separated on a D-Code Universal Mutation Detection System (Bio-Rad Laboratories, Inc. USA) with the following conditions: V3 region of bacterial 16S rRNA gene, run on a 8% polyacrylamide gel for 900 min at a constant voltage of 70 V and at 60°C in a 45~70% horizontal denaturant gradient; ITS1 gene, run on a 8% polyacrylamide gel for 780 min at a constant voltage of 70 V and at 58°C in a 30~60% horizontal denaturant gradient. The gels were stained with SYBR Green I and visualized with Molecular Imager® Gel Doc™ XR^+^ (Bio-Rad Laboratories). Similarities between banding patterns and clusters were analyzed with Quantity One software.

### Real-time PCR quantification of 16S rRNA and its genes

To estimate the abundance of bacteria and fungi, the copy numbers of bacterial 16S rRNA genes and fungal ITS1 genes were quantified using real-time PCR with primers 341F/518R (Moore et al., 2011) and 5.8S/ITS1F (Fierer et al., 2005), respectively. All the real-time PCR assays were triplicately conducted on a CFX96 Optical Real-Time Detection System (Bio-Rad Laboratories, Inc. USA). The PCR reaction mix contained 10 μl 2 × PCR buffer (iQ™ SYBR Green Supermix, Bio-Rad), 2.5 μl of each primer (2 μmol l^−1^), 1 μl of template DNA, and sterile deionized water added to 20 μl. The qPCR conditions were as follows: an initial denaturation at 95°C for 5 min; 40 cycles of denaturation at 95°C for 15 s, annealing at 56°C (bacteria) or 55°C (fungi) for 30 s. The standard curves were generated using plasmid DNA containing one V3 of bacterial 16S rRNA gene or fungal ITS1 gene. Standard template dilution series from 1.54 × 10^8^ to 1.54 × 10^2^ copies (bacteria) or 8.73 × 10^8^ to 8.73 × 10^2^ copies (fungi) per assay was used. Melting curve analysis and agarose electrophoresis were performed to confirm the specificity of amplification products. Amplification efficiencies of 90~102% were obtained, with *R*^2^ > 0.99.

### Miseq-sequencing of 16S rRNA and its genes

In order to evaluate the SMC structure with higher resolution, Miseq-sequencing of the bacterial 16S rRNA gene V3–V4 region and fungal ITS genes ITS1 region were conducted. For this, only three replicate samples were selected from four replicate samples for each treatment, according to the DGGE profiling. For bacteria and fungi, the 515F/806R (Peiffer et al., 2013) and ITS1F/ITS2 (Mueller et al., 2014) primer sets were used, with each reverse primers fused with a unique 6-mer barcode for each samples. Firstly, PCR reactions were carried out in 30 μl reactions with 15 μl of Phusion® High-Fidelity PCR Master Mix (New England Biolabs); 0.2 μmol l^−1^ of forward and reverse primers, and about 10 ng template DNA. Thermal cycling consisted of initial denaturation at 98°C for 1 min, followed by 30 cycles of denaturation at 98°C for 10 s, annealing at 50°C for 30 s, and elongation at 72°C for 60 s, finally 72°C for 5 min. Then the PCR products were electrophoresed on 2% agarose gel for detection, and samples with bright main strip between 400~450 bp were chosen for further experiments. PCR products were mixed in equal density ratios. Then, the mixture PCR products were purified with GeneJET Gel Extraction Kit (Thermo Scientific). Sequencing libraries were generated using NEB Next® Ultra™ DNA Library Prep Kit for Illumina (NEB, USA) following manufacturer's recommendations and index codes were added. The library quality was assessed on the Qubit 2.0 Fluorometer (Invitrogen) and Agilent Bioanalyzer 2100 system. Finally, the library was sequenced on an Illumina MiSeq platform and 300 bp paired-end reads were generated. Raw sequence reads were de-multiplexed, quality-filtered, processed and analyzed using QIIME (Caporaso et al., 2012). Sequences with ≥ 97% similarity were assigned to the same OUT by UCLUST clustering (Edgar, 2010). One representative sequence for each OUT were picked and used the RDP Classifier and Greengenes database (for bacterial 16S rRNA genes) and UNITE database (for fungal ITS1 genes) to annotate taxonomic information. Samples were rarified to 43,860 and 12,721 tags for bacteria and fungi, respectively, prior to downstream analyses. Because of low production of reads for SG3, we excluded this sample in the downstream analysis to avoid an underestimate with low reads number when randomly subsampling sequences to ensure a same sequence depth.

### Data analysis and statistics

Analysis of variance (ANOVA) was conducted using IBM SPSS statistics software version 21 (SPSS Inc., Chicago, IL) to test significant difference of soil chemical properties, SMC alpha diversity, and copies of bacterial 16S rRNA genes and fungal ITS genes (*n* = 4, except for sequencing data *n* = 3), and *t*-tests were also conducted for legume and grass (*n* = 12, expect for sequencing data *n* = 9). Principle component analysis (PCA) on the basis of soil chemical property was performed using IBM SPSS statistics software to reveal the influence of plants. Bacterial and fungal taxa at phylum level selected by plants were plotted in SigmaPlot 12.5. To further uncover biomarkers, linear discriminant analysis (LDA) effect size (LEfSe) was performed to find significantly abundant bacterial and fungal taxa within three groups (legume, grass and control). The factorial Kruskal-Wallis sum-rank test (α = 0.05) was used to identify taxa with significant differential abundances between categories (using the more strict all-against-all comparisons), and then LDA was performed to estimate the effect size of each differentially abundant taxon. Then significant taxa and others were used to generate taxonomic cladograms reflecting differences within groups (Segata et al., 2011). Cluster analysis of DGGE profiles and sequencing data at phylum level were analyzed and visualized using IBM SPSS statistics software and Past 3.11 to evaluate variations of soil samples with UPGMA algorithm.

### Sequence accession numbers

The 16S rRNA gene and ITS DNA sequence data were submitted to NCBI Sequence Read Archive (SRA) with accession number SRX2589894 and SRX2590037 for bacteria and fungi, respectively.

## Results

### Soil chemical properties as affected by plant species

Soil chemical analysis indicates that legume and grass affected the soil chemical properties in much different patterns. Although they did not differentially affect TOC, TP, TK, AN, AK, and C/N, their differential effects on pH, DOC, TN, AP was clearly observed (Table 1). Soil pH associated with legume was significantly lower than that associated with grass, with both higher than control. Similarly, DOC associated with legume was lower than that associated with grass. In contrast, TN and AP associated with legume were higher than those associated with grass.

**Table 1:** Soil chemical property as affected by legume or grass.

**Sample**		**pH**	**TOC (g kg^−1^)**	**DOC (mg kg^−1^)**	**TN (g kg^−1^)**	**TP (g kg^−1^)**	**TK (g kg^−1^)**	**AN (mg kg^−1^)**	**AP (mg kg^−1^)**	**AK (mg kg^−1^)**	**C/N**
Legume	SG	5.44 ab	10.3 a	174.1 ab	0.73 d	0.70 b	13.03 b	69.3 a	163.5 ab	15.6 a	14.1 a
	TP	5.59 bc	9.3 a	158.6 ab	0.61 bc	0.57 a	10.92 a	64.0 a	171.5 b	19.3 ab	15.2 a
	MS	5.72 c	9.3 a	171.0 ab	0.59 b	0.58 a	11.48 ab	71.9 a	166.5 ab	33.0 c	15.9 a
	average	5.58^**^	9.6 NS	167.9^*^	0.65^*^	0.62 NS	11.81 NS	68.4 NS	167.2^***^	22.6 NS	15.0 NS
Grass	PN	5.75 c	9.6 a	199.0 b	0.65 c	0.70 b	12.90 ab	71.2 a	158.5 a	13.3 a	14.9 a
	FA	5.75 c	8.7 a	174.1 ab	0.58 b	0.56 a	11.30 ab	69.1 a	156.8 a	16.5 ab	15.0 a
	LP	5.78 c	9.9 a	186.6 ab	0.51 a	0.56 a	11.35 ab	66.1 a	158.3 a	23.1 b	19.3 b
	average	5.76^**^	9.4 NS	186.6^*^	0.58^*^	0.61 NS	11.85 NS	68.8 NS	157.8^***^	17.6 NS	16.4 NS
Control		5.24 a	8.8 a	146.1 a	0.63 bc	0.62 ab	12.20 ab	59.6 a	166.5 ab	68.0 d	14.0 a
*F*-value of ANOVA		12.581	2.580	2.995	25.542	5.925	3.572	1.106	5.043	176.027	6.586
*P*-value		0.000	0.049	0.028	0.000	0.001	0.013	0.392	0.002	0.000	0.001

Data followed with lower letters in each column are separated by one-way ANOVA (Turkey HSD multiple range test, P = 0.05). PN, Paspalum natatum; FA, Festuca arundinacea; LP, Lolium perenne; SG, Stylosanthes guianensis; TP, Trifolium pratense; MS, Medicago sativa; Control, no plants. NS and

*, **, *** *indicate no significant difference and significant difference at P = 0.05, 0.01, 0.001, respectively, between the averages of legume and grass*.

**Figure 1:**
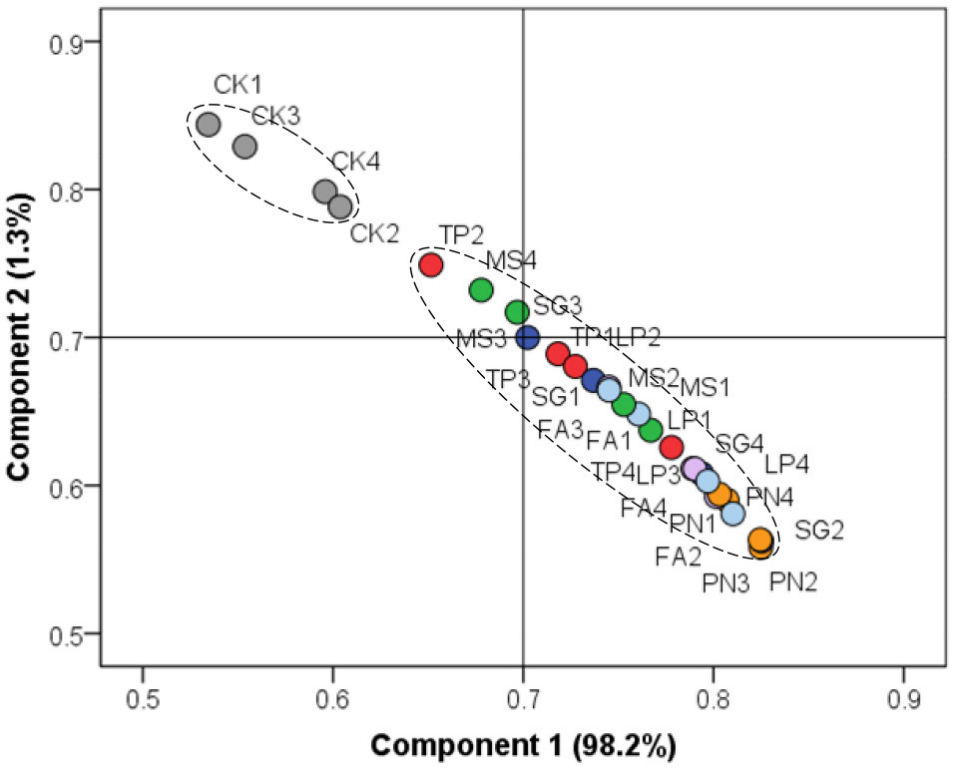
Principle component analysis on the basis of soil chemical property. PN, *Paspalum natatum*; FA, *Festuca arundinacea*; LP, *Lolium perenne*; SG, *Stylosanthes guianensis*; TP, *Trifolium pratense*; MS, *Medicago sativa*; CK, Control without plants.

On the basis of soil chemical property, we performed PCA to evaluate the similarity of effect by three legume species or three grass species against control. Figure 1 shows that the soil chemical property associated with legume or grass was different from that without plants, while the soil chemical property associated with six different plant species did not separate from each other well.

### Soil microbial community structure as affected by plant species

According to DGGE profiles, there was no significant difference in the fungal community structure between legume and grass (Table 2); however, Shannon-Weaver diversity index (*H'*), species evenness (*J*), and species richness (*S*) of the bacterial community associated with legume were significantly higher than those associated with grass. As a whole, the parameters of community structure associated with legume or grass were comparable to those of the control, although some individual species showed significant influences (Table 2).

**Table 2:** Alpha diversity of the soil microbial community associated with legume and grass on the basis of DGGE profiling.

**Sample**		***H'***	***J***	***S***
**BACTERIAL COMMUNITY**				
Legume	SG	2.99 c	0.91 c	27.25 ab
	TP	2.95 c	0.88 bc	28.29 ab
	MS	2.86 bc	0.85 abc	29.33 b
	average	2.93^***^	0.88^***^	28.29^***^
Grass	PN	2.78 abc	0.85 abc	26.53 ab
	FA	2.59 a	0.80 a	25.80 a
	LP	2.68 ab	0.82 ab	25.80 a
	average	2.68^***^	0.82^***^	26.04^***^
Control		2.88 bc	0.87 bc	27.25 ab
*F*-value of ANOVA		7.496	6.559	4.167
*P*-value		0.000	0.001	0.007
**FUNGAL COMMUNITY**				
Legume	SG	2.14 a	0.73 a	18.75 a
	TP	2.09 a	0.72 a	17.99 a
	MS	2.37 b	0.78 a	21.07 a
	average	2.20 NS	0.74 NS	19.27 NS
Grass	PN	2.17 ab	0.75 a	18.01 a
	FA	2.23 ab	0.77 a	18.01 a
	LP	2.08 a	0.72 a	18.25 a
	average	2.16 NS	0.75 NS	18.09 NS
Control		2.18 ab	0.75 a	18.50 a
*F*-value of ANOVA		5.234	2.914	2.303
*P*-value		0.002	0.032	0.073

Data followed with lower letters in each column are separated by one-way ANOVA (Turkey HSD multiple range test, P = 0.05). H', Shannon-Weaver diversity index; J, species evenness; S, species richness. PN, Paspalum natatum; FA, Festuca arundinacea; LP, Lolium perenne; SG, Stylosanthes guianensis; TP, Trifolium pratense; MS, Medicago sativa; Control, no plants. NS and

*** *indicate no significant difference and significant difference at P = 0.001, respectively, between the averages of legume and grass*.

According to the Miseq-sequencing, the alpha diversity (Shannon index) of bacterial community associated with legume was significantly higher than that associated with grass; however, the alpha diversity (OTU, Chao1, and Shannon index) of fungal community associated with legume was significantly lower than that associated with grass (Table 3).

**Table 3:** Alpha diversity of the soil microbial community associated with legume and grass on the basis of Miseq-sequencing.

**Sample**		**OTU**	**Chao1**	**Shannon index**
**BACTERIAL COMMUNITY**				
Legume	SG	1763 ab	3084.4 a	9.19 abc
	TP	1847 b	2670.6 a	9.31 c
	MS	1812 ab	2600.3 a	9.25 bc
	average	1807 NS	2785.1 NS	9.25^***^
Grass	PN	1685 a	2481.8 a	9.01 a
	FA	1772 ab	2569.9 a	9.14 abc
	LP	1810 ab	2543.4 a	9.13 ab
	average	1756 NS	2531.7 NS	9.09^***^
Control		1781 ab	2606.8 a	9.08 ab
*F*-value of ANOVA		3.056	0.674	7.647
*P*-value		0.040	0.673	0.001
**FUNGAL COMMUNITY**				
Legume	SG	351 b	453.0 ab	2.77 a
	TP	189 a	260.2 a	2.01 a
	MS	439 b	486.9 b	4.24 b
	average	326^*^	400.0^*^	3.01^***^
Grass	PN	468 b	557.6 b	4.33 b
	FA	357 b	446.5 ab	4.20 b
	LP	463 b	563.5 b	4.62 b
	average	429^*^	522.5^*^	4.38^***^
Control		378 b	489.8 b	4.09 b
*F*-value of ANOVA		15.668	5.151	26.176
*P*-value		0.000	0.005	0.000

Data followed with lower letters in each column are separated by one-way ANOVA (Turkey HSD multiple range test, P = 0.05). PN, Paspalum natatum; FA, Festuca arundinacea; LP, Lolium perenne; SG, Stylosanthes guianensis; TP, Trifolium pratense; MS, Medicago sativa; Control, no plants. NS and

*, **, *** *indicate no significant difference and significant difference at P = 0.5, 0.01, 0.001, respectively, between the averages of legume and grass*.

### The abundance of bacteria and fungi as affected by plant species

To investigate the effects of legume and grass on microbial abundance, we performed qRT-PCR targeting the 16S rRNA V4 gene and ITS gene. Both legume and grass significantly increased the bacterial and fungal abundance in soils (Table 4). When no significant difference in bacterial abundance was observed between legume and grass, the fungal abundance associated with legume was significantly higher than that associated with grass (Table 4).

**Table 4:** The abundance of bacterial 16S rRNA V4 region and fungal ITS genes in soils as affected by legume and grass.

**Sample**		**Bacteria (× 10^9^ copies/g soil)**	**Fungi (× 10^8^ copies/g soil)**	**Bacteria/fungi**
Legume	SG	7.66 d	7.96 d	10.4 ab
	TP	4.40 b	7.50 cd	5.7 a
	MS	6.64 bcd	3.44 ab	20.0 b
	average	6.23 NS	6.30^**^	12.1 NS
Grass	PN	6.96 cd	4.74 bc	14.9 ab
	FA	5.46 bcd	2.81 ab	20.3 b
	LP	5.26 bc	3.41 ab	16.9 b
	average	5.89 NS	3.65^**^	17.4 NS
Control		1.26 a	1.22 a	10.7 ab
*F*-value of ANOVA		16.887	13.723	5.948
*P*-value		0.000	0.000	0.001

Data followed with lower letters in each column are separated by one-way ANOVA (Turkey HSD multiple range test's, P = 0.05). PN, Paspalum natatum; FA, Festuca arundinacea; LP, Lolium perenne; SG, Stylosanthes guianensis; TP, Trifolium pratense; MS, Medicago sativa; Control, no plants. NS and

** *indicate no significant difference and significant difference at P = 0.01, respectively, between the averages of legume and grass*.

**Figure 2:**
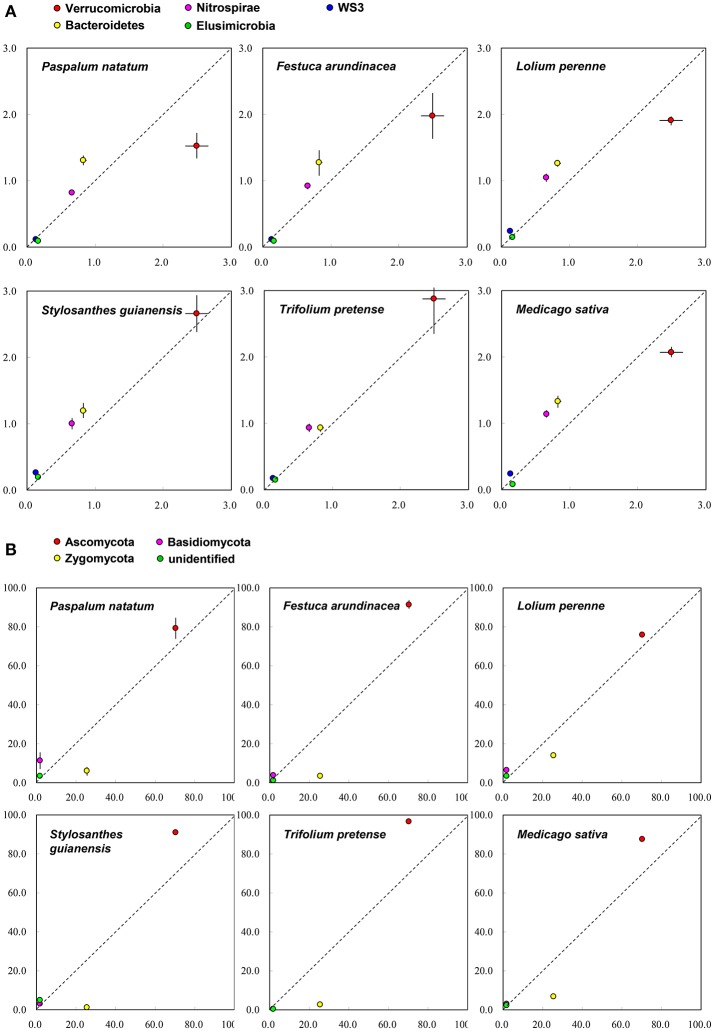
The comparison of soil microbial taxonomic profile (relative abundance, %) at phylum level between control (X-axis) and each plant species (Y-axis) on the basis of the Miseq-sequencing. **(A)** Bacteria; **(B)** Fungi.

On the basis of Miseq-sequencing, we compared the microbial abundance between control and each plant species. Figure 2A indicates that, at phylum level, all six plant species increased the abundance of *Bacteroidetes* and *Nitrospirae*. All three grass species decreased the abundance of *Verrucomicrobia*; however, two (SG and TP) out of three legume species increased it (Figure 2A), indicating the difference between legume and grass. For fungi, it is clear that all six plant species increased the abundance of *Asomycota*, but decreased the abundance of *Zygomycota* (Figure 2B).

**Figure 3:**
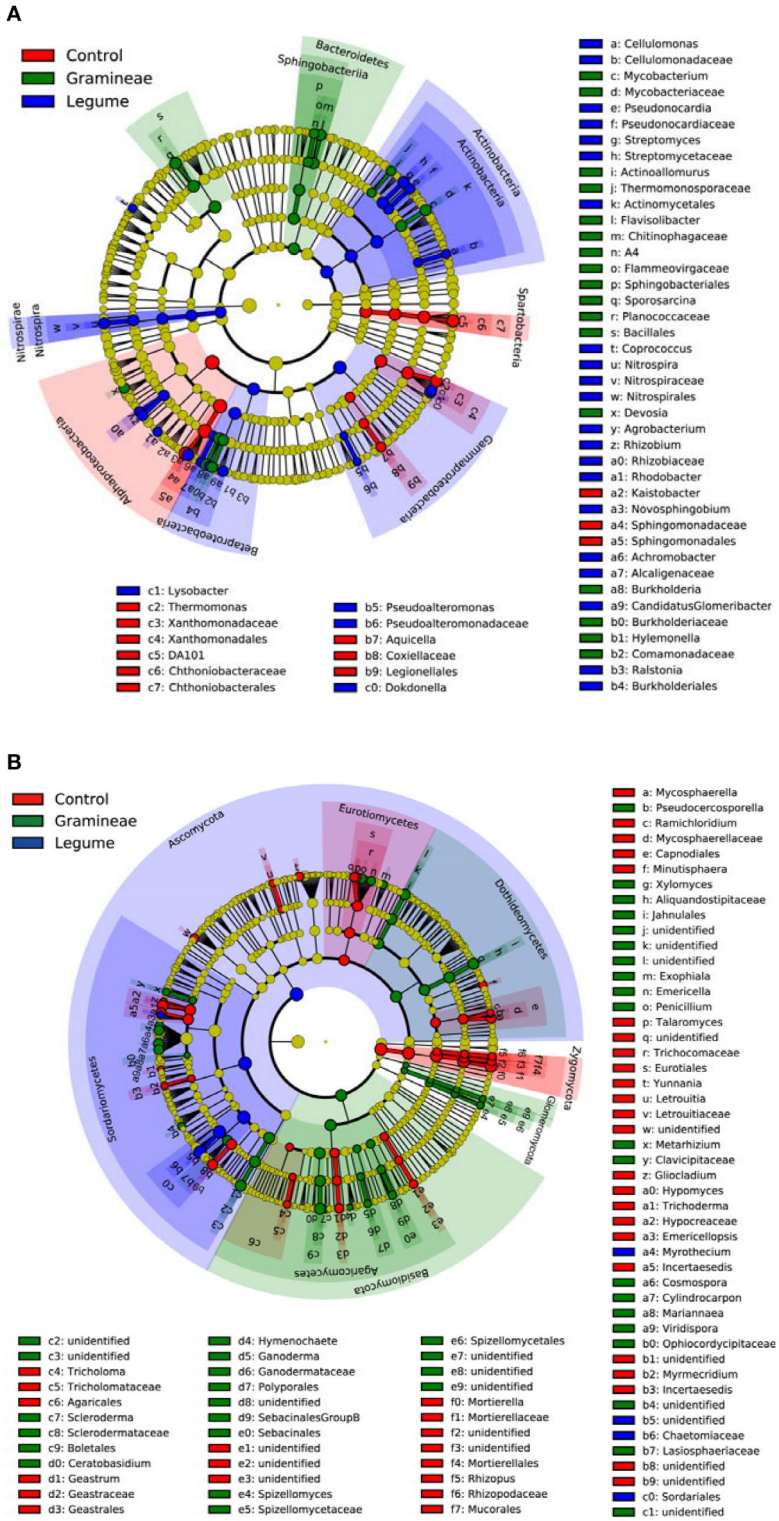
Cladogram indicating the phylogenetic distribution of microbial lineages as affected by legume and grass. Each circle's diameter is proportional to the given taxon's relative abundance; circles represent phylogenetic levels from phylum to genus inside out. Red indicates control without plants, green indicates grass, blue indicates legume, and yellow indicates non-significant. **(A)** Bacteria; **(B)** Fungi.

We performed linear discriminant analysis (LDA) to reveal the microbial biomarkers of legume and grass. For bacteria, Figure 3A shows that legume enriched *Actinobacteria* and *Nitrospirae* phyla, in contrast to *Bacteroidetes* phylum enriched by grass. In more details, legume enriched Actinobacteria, Gammaproteobacteria, Betaproteobacteria, and Nitrospira classes, and grass enriched Sphingobacteria class; while Spartobacteria and Alphaproteobacteria classes were enriched in soils without plants (Figure 3A). For fungi, Figure 3B shows that legume, grass and control enriched *Ascomycota, Basidiomycota* and *Zygomycota* phyla, respectively. In more detail, legume enriched Sordariomycetes class, and grass enriched Dothideomycetes, Glomeromycetes, Agaricomycetes; while Eurotiomycetes was enriched in soils without plants (Figure 3B). In general, these microbial taxa can be the candidate biomarkers of legume or grass.

### The variation in microbial community structure among plant species

**Figure 4:**
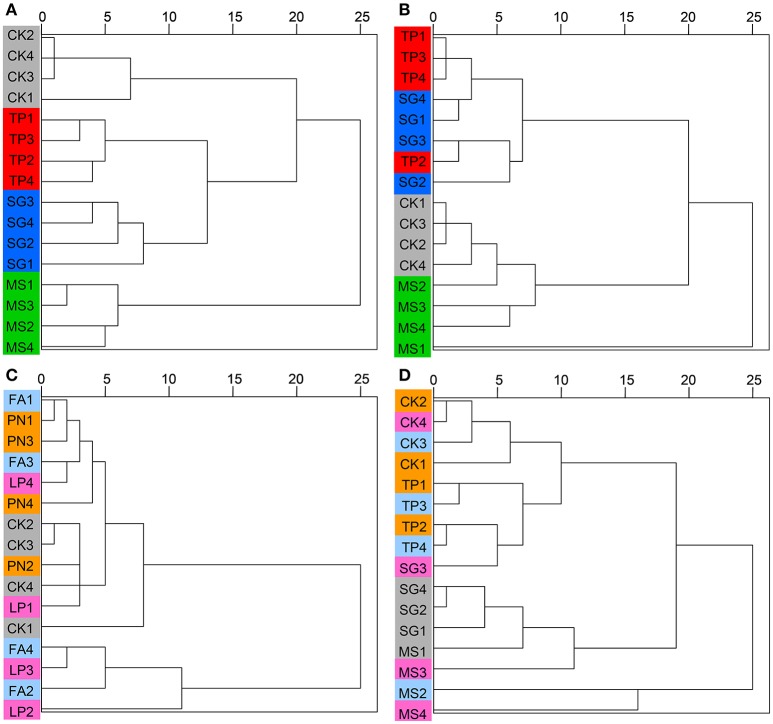
Cluster analysis of the soil microbial community associated with legumes and grasses on the basis of DGGE profiling. **(A)** Bacteria of legumes; **(B)** Fungi of legumes; **(C)** Bacteria of grasses; **(D)** Fungi of grasses. PN, *Paspalum natatum*; FA, *Festuca arundinacea*; LP, *Lolium perenne*; SG, *Stylosanthes guianensis*; TP, *Trifolium pratense*; MS, *Medicago sativa*; CK, control without plants.

**Figure 5:**
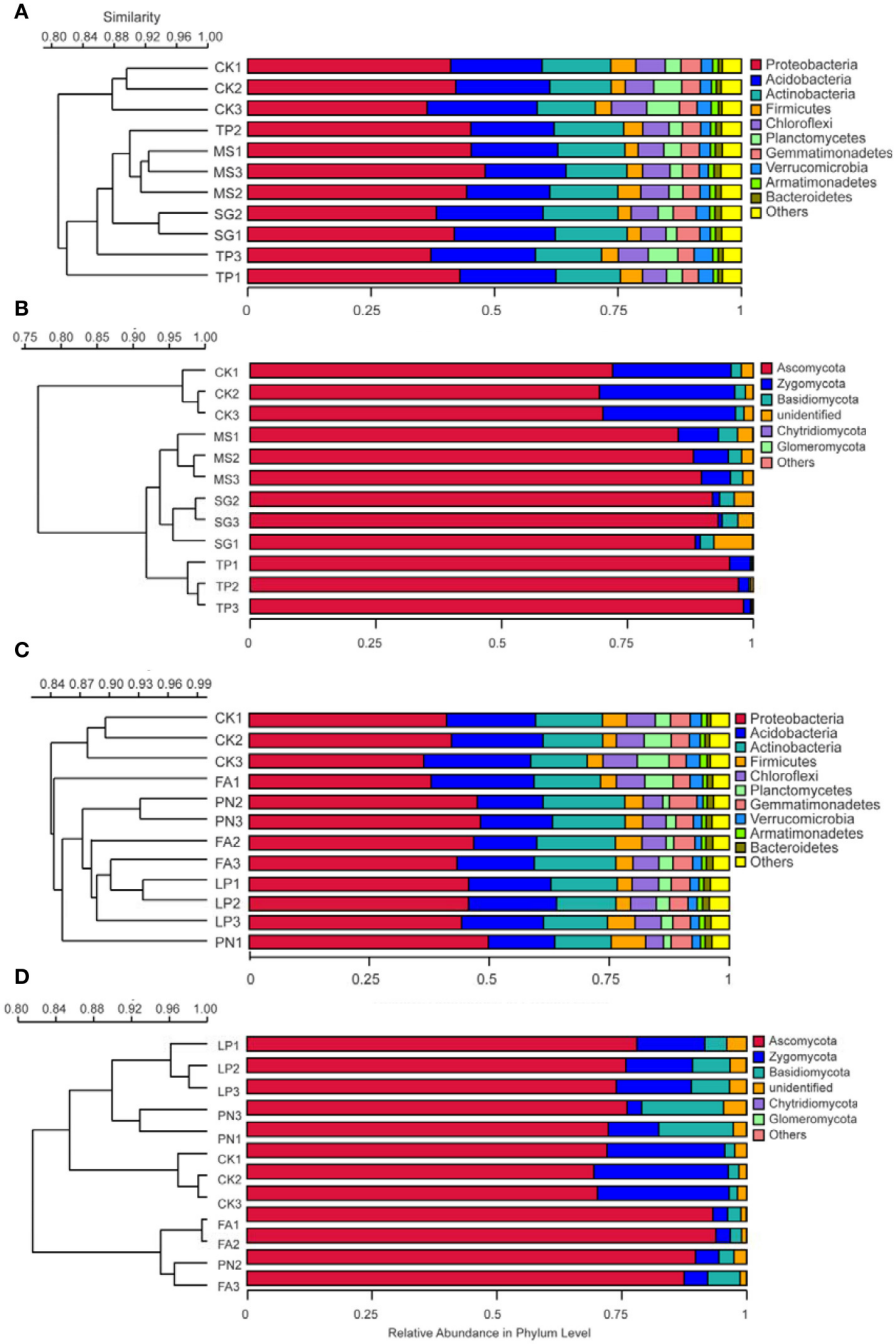
Cluster analysis of the soil microbial community associated with legumes and grasses on the basis of Miseq-sequencing. **(A)** Bacteria of legumes; **(B)** Fungi of legumes; **(C)** Bacteria of grasses; **(D)** Fungi of grasses. PN, *Paspalum natatum*; FA, *Festuca arundinacea*; LP, *Lolium perenne*; SG, *Stylosanthes guianensis*; TP, *Trifolium pratense*; MS, *Medicago sativa*; CK, control without plants.

DGGE profiles were subjected to clustering analysis to reveal the similarity of the SMC structure associated with three species of legume or grass. It is obvious that both the bacterial and the fungal community structure associated with three legume species separated from each other, and in contrast, neither the bacterial nor the fungal community structure associated with three grass species separated (Figure 4). These data suggest that the SMC associated with three legume species were much different from each other, while the SMC associated with three grass species were similar to a certain degree. Clustering analysis was also performed on the basis of Miseq-sequencing (at phylum level) to validate the result from DGGE profiling, and the similar result was obtained (Figure 5).

## Discussion

Legume and grass, as important functional groups in most plant ecosystems, are much different from each other. Despite those distinguishable differences in physiological and biochemical features (Isobe et al., 2001; Haling et al., 2016; Kidd et al., 2016; Schaller et al., 2016), their differential impacts on edaphic property were explored in this study. To represent the legume group and the grass group well, three species (each from different genera) were selected for each group. We demonstrated that legume and grass not only differentially affected the soil chemical property, but also differentially shaped the SMC structure. Moreover, we found that the variation in the SMC structure associated different legume species was greater than that associated with different grass species.

Firstly, in this study, both legume and grass increased soil pH, and the effect of grass was greater than that of legume. The beneficial effect of plants in alleviating acidity of acidic soils has been reported previously (Cui et al., 2015). Legume is less effective mainly due to the release of proton during the biological N-fixation by symbionts (Haynes, 1983). The differential uptake of different nutrient forms can also modify the soil pH (Thion et al., 2016). In addition to the lower soil pH, the DOC content was also lower in the legume soils than in the grass soils. DOC is easily decomposed by microbes and thus the measured DOC is closely related to microbial activity. According to a survey of 29 sites throughout UK, van den Berg et al. (2012) indicated that DOC was positively correlated with C/N, because decomposition is limited at high at C/N unfavorable to microbial activity. In contrast, legume increased TN and AP in this study. It is readily acknowledged that TN was increased primarily because of the biological N_2_-fixation (BNF) associated with legume (Li et al., 2015; Bichel et al., 2016; Wu et al., 2017); however, TN is not always increased by legume because the measured TN is the outcome of the balance between N input (BNF) and root N uptake. The unchanged and even decreased TN by legume was also reported previously (Sainju et al., 2017). AP is another parameter increased by legume compared with grass in this study. In this study, we used acidic soils where FePO_4_ and Al PO_4_ are the predominant inorganic P. Therefore, the release of P in the rhizosphere of legume due to the chelation of Fe and Al by organic acids can be expected because the secretion of organic acids from legume roots is normally in a larger amount than that from grass roots (Hocking, 2001; Weisskopf et al., 2008; Maltais-Landry, 2015). Moreover, the increased mineralization of organic P by legume is also possible, because phosphatase activity is higher in the rhizosphere of legume than in the rhizosphere of grass regardless of organic P addition (Li et al., 2004). Plant nutritional strategy is an important factor involved in shaping the SMC structure (Thion et al., 2016; Guyonnet et al., 2017), however, the plant biomass and nutritional traits of different species were not monitored in this study. The nutritional strategy of these species and its influence on the SMC deserves further investigation in the future.

With Miseq-sequencing and DGGE profiling, we demonstrated that legume and grass differentially shape the SMC, including community structure and composition. The differential SMC as affected by legume and grass has been reported previously. Turner et al. (2013) indicated that both the bacterial and the fungal community structure in rhizosphere were much different between pea and wheat, as revealed by comparative metatranscriptomics. With PLFA technique, Ladygina and Hedlund (2010) showed the similar result, namely an obvious difference between *Lotus corniculatus* (legume) and *Holcus lanatus* (grass). In this study, DGGE profiling indicated that the bacterial community structure (Shannon-Weaver diversity index, species evenness, species richness) associated with legume was much different from that associated with grass, and Miseq-sequencing showed that the fungal community diversity (OTU, Chao1, Shannon index) was also much different between them. The driving force underlying the differential SMC between legume and grass can be, at least, two aspects: different root exudates and different influences on soil chemical property. The different root exudates between legume and grass have been demonstrated previously. Isobe and Tsuboki (1998) and Isobe et al. (2001) compared five legume species and four grass species, and found that legume secreted more amino acids, sugar, and flavonoids than grass. These organic compounds of low molecular weight can play critical regulation role in the SMC (de Nobili et al., 2001; Michalet et al., 2013; Bakker et al., 2015; Szoboszlay et al., 2016). Moreover, Weisskopf et al. (2008) pointed out that the secretion of organic acids by lupin was also greater than that by wheat, and this difference explained a significant proportion (15%) of the difference in their associated SMC. On the other hand, in addition to the amount of exudates, the exudate profile (or composition) of legume and grass is probably much different from each other, and consequently, can be critical in regulating the SMC. However, how the root exudate profile or how a specific organic compound regulates the SMC is far from understanding. Therefore, it is necessary to put these work forward in the future.

When considering the specific microbial taxa in this study, we found that legume enriched more fungi, which was reported previously (Turner et al., 2013). At the phylum level, it seems that grass decreased but legume increased the abundance of *Verrucomicrobia*. The phylum *Verrucomicrobia* is recently defined by phylogenetic analysis of 16S rRNA gene sequences, with very few isolates cultured so far (Hedlund, 2010). Its members are a globally distributed, abundant, and active group of soil and water bacteria (Sangwan et al., 2005; Bergmann et al., 2011). Although its function profile is largely unknown due to its unculturability, its possible roles in degrading contaminants and as indicator of soil fertility have been reported (Cardman et al., 2014; Navarrete et al., 2015). At class level, our results indicate that legume enriched Actinobacteria, Gammaproteobacteria, Betaproteobacteria, Nitrospira, and Sordariomycetes, in contrast to Sphingobacteria, Dothideomycetes, Glomeromycetes, and Agaricomycetes enriched by grass. Actinobacteria play diverse roles in their associations with plants (Barka et al., 2016); therefore, the increased abundance of Actinobacteria by legume probably contributes to more comprehensive influence of legume on soil environments over grass. Nitrospira contains one family (Nitrospiraceae), and the largest genera *Nitrospira* can perform complete nitrification (Daims et al., 2015).

Interestingly, our data show that the variation in the SMC structure associated with different legume species is greater than that associated with different grass species, which is supported by the conclusion of Berg and Smalla (2009), namely, monocotyledonous grass species show highly similar rhizosphere community in diverse studies. To our knowledge, this is the first report to directly reveal the difference between legume and grass in this aspect. As aforementioned, plant can shape the SMC mainly via two mechanisms, e.g., root exudates and soil chemical property. Many work indicated that soil chemical property is the critical factor driving the SMC (Muhammad et al., 2014). For instance, soil pH was demonstrated to be the predictor of soil bacterial community structure at the continental scale (Lauber et al., 2009). Both water quantity and quality of the irrigation regulated the SMC in grapefruit orchards (Bastida et al., 2017). In drained Mediterranean peaty soils, clay, bulk density, and exchangeable calcium drove ammonia-oxidizing community, while silt, ammonium, and exchangeable potassium drove *nirK*-type denitrifier community (Ciccolini et al., 2016). In this study, three legume species also differentially affected some soil chemical parameters, such as TN, TP, TK, AK, however, PCA indicated that the soil chemical property as a whole did not differ much among them. It is likely that other factors in addition to the variation in the soil chemical property also contributed to the significant segregation of the SMC associated with three legume species. It is the case for three grass species. Consequently, root exudates are probably the primary cause responsible for the difference between legume and grass, that is, the root exudate profile differs much among three legume species, while its does not among three grass species. The root exudate profiles were not characterized in this study; however, it is well acknowledged that flavonoids in the root exudates of legume differ much among different species (Gomaa et al., 2015). It is also the case for the amino acids and organic acids in root exudates. For example, citrate was detectable only in the root exudates of white lupin, but not of field pea and faba bean (Nuruzzaman et al., 2006), and the change patterns of carboxylate in rhizosphere were different among even three cultivars of chickpea (Wouterlood et al., 2004). It is acknowledged that the distribution patterns of secondary metabolites (including non-protein amino acids, organic acids, phenolics) in legume species do not agree with the phylogeny of the species producing them, probably due to convergent evolution, a contribution of endophytic fungi, or the horizontal gene transfer from bacteria (see review by Wink, 2013, and literature therein). Szoboszlay et al. (2016) indicated that different flavonoids (7,4′-dihydroxyflavone and naringenin) differentially regulated the microbial community. How the amino acid profile and organic acid profile affect the microbial community is largely unknown, and more work in the future is expected. Moreover, legume roots normally release much more organic compounds than grass roots, and thus, it is unquestionable that a large amount of root exudates with species-specific profile shapes the unique SMC for each legume species, but not for grass species.

## Author contributions

Designed experiments: YZ, HZ, and QY. Performed experiments: YZ. Analyzed results: YZ, SF, and QY. Wrote the manuscript: YZ, HZ, and QY.

### Conflict of interest statement

The authors declare that the research was conducted in the absence of any commercial or financial relationships that could be construed as a potential conflict of interest.
